# Adjuvant and Neoadjuvant Treatments for Resectable Hepatocellular Carcinoma

**DOI:** 10.1007/s11912-023-01455-9

**Published:** 2023-09-09

**Authors:** Christiana J. Crook, Daneng Li

**Affiliations:** Department of Medical Oncology and Therapeutics Research, City of Hope Comprehensive Cancer Center, 1500 E Duarte Road, Duarte, CA 91010 USA

**Keywords:** Hepatocellular carcinoma, Resectable, Neoadjuvant, Adjuvant, Liver-directed treatment, Systemic treatment

## Abstract

**Purpose of Review:**

This review provides an update on the developments of adjuvant and neoadjuvant liver-directed and systemic therapy options for patients with resectable hepatocellular carcinoma.

**Recent Findings:**

Data on liver-directed treatment in the adjuvant and neoadjuvant settings are sparse and results are conflicting; many studies suggest that optimizing patient selection criteria is a key milestone required to improve study design and clinical benefit to patients. Systemic treatment options are primarily focused on investigation of anti-PD-1/L1 immunotherapeutic agents, either alone or in combination with other drugs. Numerous clinical trials in both adjuvant and neoadjuvant settings are in progress.

**Summary:**

Exploration of liver-directed and systemic treatment options for adjuvant and neoadjuvant treatment of patients with resectable hepatocellular carcinoma has the potential to improve clinical outcomes for this patient population.

## Introduction

Liver cancer is the sixth-leading cancer diagnosis and the third-leading cause of cancer-related death worldwide; hepatocellular carcinoma (HCC) is the most common type of liver cancer, comprising 75–85% of cases [1]. Advances in imaging and surveillance techniques have increased the number of patients who can be diagnosed at earlier stages, allowing them to potentially receive curative treatment [2, 3]. Surgical resection, transplantation, and ablation comprise current curative treatment options for patients with HCC [2, 4]. However, the risk of recurrence after curative treatment is high, with 5-year recurrence rates ranging from 40 to 70% [5]. Furthermore, there are no FDA-approved treatments to decrease the risk of disease recurrence. Although there have been significant advances in the systemic treatment field for patients with advanced unresectable HCC [2], treatments for patients with resectable HCC are limited to physicians’ discretion or clinical trials. This gap in knowledge has resulted in a significant unmet clinical need for this patient population.

Adjuvant and neoadjuvant treatments have been utilized for many years in a variety of cancers, such as breast [6, 7], colon [8–11], and pancreas [12–14]. These treatments have been shown to reduce risk of disease recurrence and increase length of survival and are recommended treatment options by the National Comprehensive Cancer Network [15–17]. Given the promising results demonstrated for adjuvant and neoadjuvant treatment for patients with other types of resectable cancers, the role of adjuvant and neoadjuvant treatment for patients with resectable HCC is a promising field of study.

In this review we report developments in adjuvant and neoadjuvant (including perioperative) treatment for patients with resectable HCC, with a focus on liver-directed treatments and systemic treatments. We conclude with a look at ongoing clinical trials that have the potential to improve outcomes for this patient population.

## Adjuvant Treatment

### Liver-Directed Treatment

Liver-directed treatment is the preferred treatment modality for patients with intermediate-stage HCC or early-stage HCC for whom curative options are not feasible or unsuccessful [4]. The role of liver-directed treatment in the adjuvant setting has not been well-investigated. Most of the studies on adjuvant liver-directed treatment discussed in this review were performed in China, which may limit generalizability of their results.

The utility of adjuvant transarterial chemoembolization (TACE) was investigated by Wang et al. in a phase 3 single-center randomized controlled trial [18]. Patients with hepatitis B virus (HBV)–related HCC who underwent curative resection and were at intermediate risk (single tumor > 5 cm without microvascular invasion) or high risk (single tumor with microvascular invasion or two or three tumors) of recurrence received adjuvant TACE or no treatment (control). Median recurrence-free survival (RFS) was 49.5 months (95% confidence interval [CI] 37.2–61.8) for TACE compared to 23.8 months (95% CI 15.7–31.9) for control. Subgroup analyses confirmed a clinical benefit with adjuvant TACE across all groups, including patients with alpha fetoprotein > 20 ng/mL and patients at high risk of disease recurrence. Treatment was well-tolerated, with no adverse events (AEs) ≥ grade 3. The benefit of adjuvant TACE was further supported by Wu et al. in a retrospective propensity score matching analysis of patients who underwent hepatectomy and received adjuvant TACE or no treatment (control) [19]. Patients who received adjuvant TACE had improved 3-year disease-free survival (DFS) and overall survival (OS) compared to control patients. However, patients at low risk of disease recurrence (single tumor ≤ 5 cm with no satellite nodules or micro/macrovascular invasion) after curative resection may not benefit from adjuvant TACE [20]. A retrospective propensity score matching analysis by Feng et al. found that low-risk patients who received adjuvant TACE had reduced mean RFS (51.0 ± 4.8 months) compared to patients who did not receive adjuvant TACE (66.0 ± 2.4 months); there were no differences in OS [20]. Identification of patients likely to derive significant clinical benefit from adjuvant TACE is paramount to better refine treatment plans and avoid unnecessary treatment. To address this issue, Liang et al. developed an online calculator to predict survival benefit with or without adjuvant TACE for patients who have undergone surgical resection [21]. The calculator incorporates eight recurrence risk factors (resection margin, tumor size/number, micro/macrovascular invasion, portal hypertension, alpha fetoprotein expression, and Child–Pugh score) and provides estimates for net improvement in survival with or without adjuvant TACE. The authors emphasize that the calculator is meant to provide guidance and serve as a decision aid for treating physicians; it is not meant to provide absolute treatment recommendations. While there are promising data for the use of adjuvant TACE among patients at intermediate or high risk of recurrence, further prospective studies are needed to better investigate its role in this patient population.

Another option for adjuvant liver-directed treatment is stereotactic body radiotherapy (SBRT). Shi et al. performed a single-center randomized controlled trial among patients with BCLC stage 0 or A HCC who underwent hepatectomy with marginal resection with or without adjuvant SBRT [22]. Patients who received SBRT had improved 5-year DFS compared to patients who received surgery alone (56.1% vs 26.3%, p = 0.005). However, there was no significant difference in 5-year OS (75.0% vs 53.7%, p = 0.053). The role of adjuvant SBRT requires further exploration.

### Systemic Treatment

The tyrosine kinase inhibitor (TKI) sorafenib was the standard of care first-line systemic treatment option for patients with advanced unresectable HCC from 2007 to 2018 [2]. The phase 3 randomized, double-blind, placebo-controlled STORM trial investigated adjuvant sorafenib compared to placebo in HCC patients who received curative resection or ablation with complete radiologic response at intermediate risk (single tumor > 2 cm, well- or moderately-differentiated, and no microvascular invasion or satellite tumors) or high risk (microvascular invasion, satellite tumors, poorly-differentiated, or two or three tumors (each ≤ 3 cm)) of recurrence [23]. Patients received 400 mg oral sorafenib or placebo twice daily for up to four years or until disease recurrence. Median RFS was 33.3 months (95% CI 27.6–44.0) for patients who received sorafenib and 33.7 months (95% CI 27.6–39.0) for patients who received placebo (hazard ratio [HR] 0.940, 95% CI 0.780–1.134, one-sided p = 0.26). There were no significant differences between sorafenib and placebo for time to recurrence (HR 0.891, 95% CI 0.735–1.081, one-sided p = 0.12) or OS (HR 0.995, 95% CI 0.761–1.300, one-sided p = 0.48). The authors concluded that sorafenib is not a recommended adjuvant treatment. However, Huang et al. performed a meta-analysis comprising 13 studies of 2655 patients and reported that adjuvant sorafenib may improve RFS and OS and reduce recurrence rates [24]. Of note, 11 of the 13 studies were conducted in China; the authors caution that additional studies in a broader patient population are needed to further investigate the role of adjuvant sorafenib.

Lin et al. performed a retrospective propensity score matching analysis comparing patients at high risk (tumor > 5 cm with microvascular invasion, ≥ 3 tumor nodules, ruptured HCC, or tumor thrombus in the portal vein, hepatic vein, or bile duct) of recurrence post-hepatectomy who received adjuvant TACE with or without a TKI (sorafenib, lenvatinib, or rivoceranib (apatinib)) [25]. Patients who received TACE with a TKI had improved 2-year DFS compared to patients who received TACE alone (20.9% vs 12.2%, p = 0.01). Although this study did not compare TKI plus TACE versus TKI alone, it suggests that use of TKIs in the adjuvant setting may be better in combination with other approaches.

Given the inconclusive results for single-agent tyrosine kinase inhibition, others have investigated different mechanisms of action for systemic adjuvant treatment. Sun et al. investigated the VEGFR2 inhibitor rivoceranib (apatinib) in patients who received curative-intent resection and had portal vein tumor thrombosis in an open-label, single-center phase 2 trial [26]. Patients received 500 mg oral rivoceranib daily in 28-day cycles until disease recurrence or intolerable toxicity. Among 30 patients who received rivoceranib, median RFS was 7.6 months (95% CI 5.7–9.5); 1-year OS was 93.3%. Treatment-related grade 3–4 AEs occurred in 46.7% of patients and there were no treatment-related deaths. The authors concluded that rivoceranib is tolerable, with preliminary evidence of tumor control. However, given its relatively low median RFS, current studies are investigating adjuvant rivoceranib in combination with the anti-PD-1 antibody camrelizumab (NCT05367687, NCT04639180). Camrelizumab and rivoceranib have been studied primarily in Asia; the recently published phase 3 CARES-310 study of camrelizumab plus rivoceranib versus sorafenib as first-line treatment in patients with unresectable HCC included only 17% non-Asian patients in each treatment arm [27]. Therefore, the efficacy and safety of camrelizumab and rivoceranib in a broader non-Asian patient population remains unclear.

The phase 3 randomized, open-label IMbrave050 trial compared adjuvant atezolizumab plus bevacizumab versus active surveillance in HCC patients who underwent curative resection or ablation and were at high risk (tumor > 5 cm, > 3 tumors, micro/macrovascular portal vein invasion (Vp1/Vp2), or Grade 3 or 4 tumor differentiation (poorly-differentiated)) of disease recurrence [28, 29••, 30••]. Patients received 1200 mg of intravenous atezolizumab plus 15 mg/kg body weight of bevacizumab every 21 days for up to 17 cycles/12 months of treatment or until disease recurrence or unacceptable toxicity. Independent review facility–assessed RFS was significantly longer in the atezolizumab/bevacizumab arm (not estimable, 95% CI 22.1 months–not estimable) compared to active surveillance (not estimable, 95% CI 21.4 months–not estimable) (HR 0.72, 95% CI 0.56–0.93, p = 0.012). The risk of disease recurrence was lower in the atezolizumab/bevacizumab arm compared to active surveillance (HR 0.67, 95% CI 0.52–0.88, p = 0.003), and there were no significant differences in patient-reported outcomes between treatment arms. Based on these results, atezolizumab plus bevacizumab is a promising adjuvant treatment for HCC patients at high risk of recurrence. Given the positive results with atezolizumab plus bevacizumab for patients with unresectable HCC [31, 32], it will be interesting to see if combination anti-PD-1 immunotherapy plus angiogenesis inhibition can also improve outcomes for HCC patients with resectable disease.

A novel target for HCC treatment is the transmembrane protein CD147, which may promote tumor growth and metastasis [33, 34]. Li et al. performed a phase 2 randomized controlled trial in patients with resected CD147-positive HCC [35•]. Patients received either adjuvant transarterial ^131^I-metuximab, a radiolabeled anti-CD147 antibody, or no adjuvant treatment (control). Recurrence-free survival at 5 years was higher for patients who received ^131^I-metuximab compared to control (43.4% vs 21.7%, HR 0.49, 95% CI 0.34–0.72, p < 0.0001). Treatment was well-tolerated, with just 9% of patients reporting grade 3–4 AEs. These results suggest that adjuvant ^131^I-metuximab for patients with CD147-positive HCC may reduce risk of recurrence. However, metuximab for treatment of unresectable HCC is only approved in China. The roles of CD147 and metuximab for HCC patients in other regions require further research.

### Ongoing Trials

Table 1 shows selected clinical trials investigating adjuvant treatment options for patients with resectable HCC. A variety of adjuvant treatment options are being explored, including immunotherapy, tyrosine kinase inhibitors, and combination treatments of systemic and/or liver-directed treatments. In particular, results from the phase 3 studies CheckMate 9DX (nivolumab versus placebo, NCT03383458), KEYNOTE-937 (pembrolizumab versus placebo, NCT03867084), and EMERALD-2 (durvalumab with or without bevacizumab versus placebo, NCT03847428) are highly anticipated. These studies together will better define whether single agent anti-PD-1/L1 immunotherapy is sufficient to result in positive outcomes in resected HCC patients or if the addition of VEGF inhibition is required for demonstrable clinical benefit.

**Table 1:** Ongoing clinical trials for adjuvant treatment of patients with resectable HCC

ClinicalTrials.gov Identifier	Phase	Intervention	Patient Population	Primary Outcomes	Estimated Study Completion Date
NCT04418401	1	Donafenib + toripalimab	Post-curative resection and at high risk of recurrence	1-year RFS rate	June 2024
NCT05367687	2	Camrelizumab + rivoceranib vs camrelizumab	Post-curative resection or ablation and at high risk of recurrence	RFS per investigator	April 2026
NCT05407519	2	Tislelizumab + sitravatinib	Post-curative resection and at high risk of recurrence	2-year RFS rate	June 2026
NCT04981665	2	TACE followed by tislelizumab	Post-curative resection and at high risk of recurrence	2-year RFS rate	December 2024
NCT04213118	2	TACE followed by anlotinib	Post-curative resection and at high risk of recurrence	Disease-free survival	September 2023
NCT03383458	3	Nivolumab vs placebo	Post-curative resection or ablation and at high risk of recurrence	RFS	December 2025
NCT03867084	3	Pembrolizumab vs placebo	Complete radiological response after surgical resection or local ablation	RFS per BICR, overall survival	August 2029
NCT03847428	3	Durvalumab ± bevacizumab vs placebo	Post-curative resection or ablation and at high risk of recurrence	RFS per BICR (durvalumab + bevacizumab vs placebo)	August 2025
NCT04639180	3	Camrelizumab + rivoceranib vs active surveillance	Post-curative resection or ablation and at high risk of recurrence	RFS per blinded independent review committee	July 2024

*BICR* blinded independent central review, *RFS* recurrence-free survival, *TACE* transarterial chemoembolization

## Neoadjuvant/Perioperative Treatment

### Liver-Directed Treatment

The role of neoadjuvant TACE for HCC patients planned for resection or transplantation is highly controversial. A retrospective study by Lee et al. looked at patients who received neoadjuvant TACE or no treatment followed by resection; patients who received neoadjuvant TACE had similar recurrence rates (35.90% vs 29.36%, p = 0.955), reduced OS (47.05% vs 52.46%, p = 0.025), and higher rates of re-hospitalization within 6 months of surgery (33.3% vs 20.8%, p = 0.011) compared to resection-only patients [36]. Amisaki et al. reported similar results from a retrospective case–control study; patients who received neoadjuvant TACE had significantly worse RFS (p = 0.043) and OS (p = 0.014) compared to resection-only patients up to 5 years post-resection [37]. Yeh et al. found no significant difference in OS (p = 0.059) or DFS (p = 0.141) between patients who received neoadjuvant TACE followed by curative-intent resection or ablation and those who received curative-intent treatment alone [38]. Dorcaratto et al. showed that neoadjuvant TACE followed by liver transplant provided no clinical benefit in terms of 5-year DFS (70% vs 63%, p = 0.454) or OS (70% vs 65%, p = 0.532) compared to transplant alone, particularly among patients who waited less than 6 months for transplant [39]. Similar results were reported by Li et al., who found that neoadjuvant TACE prior to transplant was associated with lower median OS (51.857 vs 80.930 months) and DFS (50.386 vs 80.281 months) compared to transplant-only patients [40]. While all of these studies are retrospective and have relatively small sample sizes, the consistency between results provides support for each of the authors’ conclusions that neoadjuvant TACE does not provide clinical benefit to patients with resectable HCC and in some cases may be detrimental.

On the other hand, some studies suggest that neoadjuvant TACE may improve outcomes for patients. Notably, a phase 3 randomized trial by Fang et al. reported improvements in OS and progression-free survival for patients who received neoadjuvant TACE followed by hepatectomy compared to patients who did not receive preoperative TACE [41]. Patients received TACE at least twice; patients who recorded stable disease, partial response, or complete response to TACE per modified Response Evaluation Criteria in Solid Tumors (RECIST) proceeded to resection. Patients who received neoadjuvant TACE had significantly greater 3-year OS (HR 0.3602, 95% CI 0.1914–0.6779, p = 0.0011) and progression-free survival (HR 0.4525, 95% CI 0.2891–0.7082, p = 0.0003) compared to resection-only patients. These improvements were consistent between BCLC A and BCLC B patients. For patients listed for liver transplant, a meta-analysis by Butcher et al. of 21 retrospective studies reported no significant differences in 5-year OS (HR 1.02, 95% CI 0.80–1.31, p = 0.88) or DFS (HR 1.18, 95% CI 0.73–1.88, p = 0.50) between patients who received pre-transplant TACE and patients who received transplant only [42]. However, the authors noted that patients who received neoadjuvant TACE were more likely to have larger tumor diameters at baseline and wait longer for transplant, both of which could have negatively impacted clinical outcomes. The authors conclude that neoadjuvant TACE may be used in patients with poor prognostic features, emphasizing the importance of proper patient selection.

Others have identified potential factors that could be used to select patients who may derive benefit from neoadjuvant TACE. Mo et al. performed a multicenter retrospective study of patients who received surgical resection with or without preoperative TACE [43]. Upon multivariate analysis, they found that patients with large tumor diameters (≥ 10 cm) who received preoperative TACE had better RFS (HR 0.419, 95% CI 0.269–0.652, p < 0.005) and OS (HR 0.448, 95% CI 0.260–0.773, p = 0.004) compared to patients who did not receive preoperative TACE; among patients with smaller tumor diameters (5.0–9.9 cm) there were no differences in RFS (HR = 1.045, 95% CI 0.71–1.538, p = 0.823) or OS (HR 0.961, 95% CI 0.601–1.537, p = 0.869). Li et al. reported similar findings in a multicenter retrospective propensity score matching analysis of patients with maximum HCC tumor size ≥ 10 cm who underwent curative liver resection with or without preoperative TACE [44]. Patients who received preoperative TACE had longer median OS (32.8 vs 18.1 months, p = 0.023) and RFS (12.9 vs 4.1 months, p = 0.009) compared to resection-only patients. On multivariate analysis, preoperative TACE was a significant contributor to OS (HR 1.565, 95% CI 1.083–2.262, p = 0.017) and RFS (HR 1.550, 95% CI 1.101–2.180, p = 0.012). Optimization of patient selection for neoadjuvant liver-directed therapy is needed.

Few studies have investigated neoadjuvant TACE in combination with systemic treatment. A phase 3 randomized controlled double-blind study by Hoffmann et al. compared neoadjuvant TACE plus sorafenib or placebo in patients listed for transplantation [45]. Patients who received TACE plus sorafenib had no significant differences in time to progression (71 days vs 85 days, HR 1.106, 95% CI 0.387–3.162), progression-free survival (HR 1.259, 95% CI 0.485–3.270), objective response rate (20.8% vs 26.9%), or disease control rate (66.7% vs 73.1%) compared to patients who received TACE plus placebo. A retrospective multicenter propensity score matching analysis by Wu et al. investigated neoadjuvant TACE plus lenvatinib plus anti-PD-1 immunotherapy (sintilimab, camrelizumab, tislelizumab, pembrolizumab, or toripalimab) in patients planned for resection at high risk of disease recurrence (portal vein tumor thrombus, tumor > 10 cm with tumors close to vasculature that cause resection margin < 1 cm, and > 3 tumors with one tumor > 5 cm) [46]. They concluded that combination neoadjuvant treatment improved OS and DFS; however, comparisons between TACE plus systemic treatment versus TACE alone were not performed. It remains unclear whether TACE combined with systemic therapy provides greater benefit than either treatment modality given alone; prospective studies are needed.

Hepatic arterial infusion chemotherapy (HAIC) is another option for neoadjuvant liver-directed treatment. Oyama et al. performed a single-blind randomized controlled trial comparing patients treated with or without neoadjuvant HAIC followed by radiofrequency ablation [47]. There was no significant difference in overall RFS with neoadjuvant HAIC compared to ablation only (HR 0.597, 95% CI 0.320–1.091, p = 0.094). However, there was a significant improvement in distant RFS with neoadjuvant HAIC (HR 0.468, 95% CI 0.235–0.896, p = 0.022). A retrospective study by Pan et al. investigated the effect of neoadjuvant HAIC versus adjuvant portal vein perfusion chemotherapy on survival in patients with resectable HCC [48]. Neoadjuvant HAIC was associated with improved OS (not reached vs 19.47 months, p = 0.043), intrahepatic progression-free survival (37.57 vs 6.73 months, p = 0.049), and extrahepatic metastasis-free survival (not reached vs 7.03 months, p = 0.040) compared to adjuvant portal vein perfusion chemotherapy. Of note, HAIC is currently used and investigated only in Asia; its utility in other global regions is unknown.

### Systemic Treatment

Based on promising results for immunotherapy in the treatment of patients with unresectable advanced HCC [31, 49–51], the use of neoadjuvant/perioperative immunotherapy for patients with resectable HCC is of great interest. Ho et al. performed a single-arm phase 1b study investigating neoadjuvant nivolumab plus cabozantinib in patients with locally advanced unresectable HCC [52•]. Patients received 40 mg oral cabozantinib daily for 8 weeks and 240 mg nivolumab intravenously once every 2 weeks (starting 2 weeks after initiation of cabozantinib) for 4 doses; they were restaged 2 weeks after the end of therapy and those patients deemed eligible for surgery were scheduled for surgery at least 28 days after the last dose of cabozantinib. Of 15 patients enrolled in the study, 14 completed therapy and were evaluated for surgical resection; 12 patients underwent successful surgical resection. At the pre-surgical evaluation, 13 of 14 patients had stable disease per RECIST 1.1 and 1 patient had partial response. Upon surgery, 5 of 12 patients had a complete or major (≥ 90% tumor necrosis) pathological response. The study met its primary endpoint of feasibility and supported further research of neoadjuvant systemic treatment for patients with resectable HCC.

The role of perioperative systemic treatment for patients with resectable disease was explored by Kaseb et al. in a single-center, randomized, open-label phase 2 trial [53•]. Patients received 3 doses of 240 mg intravenous nivolumab with or without 1 dose of 1 mg/kg intravenous ipilimumab before partial hepatectomy, followed by 480 mg nivolumab every 4 weeks with or without up to 4 doses of 1 mg/kg ipilimumab every 6 weeks for up to 2 years after surgery or until disease progression or intolerable toxicity. At completion of neoadjuvant treatment, 3 of 13 patients who received single-agent nivolumab had a partial response per RECIST 1.1; there were no objective responses among the 14 patients who received nivolumab plus ipilimumab. After surgical resection, median time to progression was 9.40 months for patients on the nivolumab arm and 19.53 months for patients on the nivolumab/ipilimumab arm (HR 0.89, 95% CI 0.31–2.54, p = 0.83). The incidence of grade 3–4 treatment-related AEs was higher in the combination treatment arm (6 of 14 patients vs 3 of 13 patients); however, AEs did not contribute to any delays in surgery. Three patients in each arm had a major pathological response to neoadjuvant treatment; none of these patients had developed recurrence after a median follow-up of 26.8 months. The authors concluded that perioperative immunotherapy is safe and tolerable among patients with resectable HCC and propose that future studies investigate the efficacy of perioperative nivolumab with or without ipilimumab.

Marron et al. further investigated perioperative single-agent immunotherapy in a single-arm, open-label phase 2 study in patients with resectable HCC using the anti-PD-1 antibody cemiplimab [54•]. Patients received 350 mg intravenous cemiplimab every 3 weeks for 2 cycles, followed by resection and 8 cycles of 350 mg cemiplimab every 3 weeks; the adjuvant section of the trial is not yet complete. Of 21 patients who received neoadjuvant cemiplimab, 20 proceeded to surgical resection; 15% of resected patients had a partial response per RECIST 1.1, with the rest reporting stable disease. Analysis of tumor necrosis at surgery found that 7 patients had tumor necrosis ≥ 50%, including 3 patients with 100% tumor necrosis. There were two grade 3 treatment-related AEs and no grade 4 or 5 AEs. The authors note that their duration of neoadjuvant treatment was shorter than that of Kaseb et al. [53•], which may have contributed to the lower AE incidence and higher number of patients who proceeded to surgery. They conclude that further investigation is needed to identify optimal treatment regimens and trial designs for this new treatment arena.

Combination camrelizumab plus rivoceranib (apatinib) was shown to be superior to sorafenib in terms of improving progression-free survival and OS in the first-line setting for patients with unresectable HCC [27]. This combination was explored by Xia et al. in the perioperative setting [55•]. In this single-arm, open-label phase 2 study patients with resectable HCC received 200 mg intravenous camrelizumab every 2 weeks for 3 cycles plus 250 mg oral rivoceranib (apatinib) for 21 days (starting with the first dose of camrelizumab). Surgical resection occurred 46 days after initiation of camrelizumab. Within 4 weeks post-surgery, patients received 200 mg camrelizumab every 3 weeks plus 250 mg rivoceranib daily for up to 8 cycles or until disease progression or intolerable toxicity. Of 18 patients who received neoadjuvant treatment, 17 received surgery and 13 received adjuvant treatment. Three of 18 patients recorded partial response to neoadjuvant treatment per RECIST 1.1 (16.7% objective response rate) and 14 patients had stable disease (94.4% disease control rate). After surgery, 3 of 17 patients had a major pathologic response to neoadjuvant treatment (17.6%); 1 patient had a complete pathological response (5.9%). Grade ≥ 3 treatment-related AEs occurred in 3 of 18 patients in the neoadjuvant setting and in 5 of 13 patients in the adjuvant setting; no grade 5 events occurred in the perioperative period. At 1 year post-surgery, RFS was 53.85% (95% CI 24.77–75.99). The authors encourage future studies to consider the selection of patients for neoadjuvant treatment based on the extent of disease at baseline and the selection of patients for adjuvant treatment based on pathological response to neoadjuvant treatment. An additional issue to consider is the length and timing of neoadjuvant treatment and surgery; the authors waited 3 weeks after completion of neoadjuvant rivoceranib before surgery but found that surgical resection was challenging and the rate of biliary leakage post-resection was high (3 of 17 patients). The role of tyrosine kinase inhibition in the neoadjuvant setting may require adjustments to improve the surgical experience and outcomes.

### Ongoing Trials

Table 2 shows selected clinical trials investigating neoadjuvant and perioperative treatment options for patients with resectable HCC. Of note, some studies are specifically focusing on neoadjuvant treatment for patients listed for liver transplant. A phase 2 study is investigating neoadjuvant durvalumab plus tremelimumab (NCT05027425) and a phase 3 study is investigating neoadjuvant donafenib plus TACE followed by adjuvant donafenib (NCT05576909). Other studies of interest include the recently-launched phase 1b/2 Morpheus-NEO HCC trial (neoadjuvant atezolizumab plus bevacizumab versus atezolizumab plus bevacizumab plus tiragolumab versus bevacizumab plus tobemstomig, NCT05908786), the phase 2 PRIME-HCC study (neoadjuvant nivolumab plus ipilimumab, NCT03682276), and numerous studies investigating neoadjuvant/perioperative camrelizumab in combination with rivoceranib and/or TACE (NCT04930315, NCT04701060, NCT05613478, NCT04521153).

**Table 2:** Ongoing clinical trials for neoadjuvant/perioperative treatment of patients with resectable HCC

ClinicalTrials.gov Identifier	Phase	Intervention	Patient Population	Primary Outcomes	Estimated Study Completion Date
NCT04857684	1	SBRT followed by atezolizumab + bevacizumab	Treatment-naïve resectable disease	Grade 3–4 treatment-related AEs	December 2025
NCT05225116	1	Lenvatinib + sintilimab + radiotherapy	Resectable disease with portal vein tumor thrombus	Grade ≥ 3 treatment-related AEs, number of patients who complete treatment and proceed to surgery	December 2025
NCT05185531	1	Tislelizumab + SBRT	Resectable disease	Delay to surgery, objective response rate after neoadjuvant treatment, pathologic response rate, treatment-emergent AEs	December 2024
NCT05908786	1b/2	Atezolizumab + bevacizumab ± tiragolumab vs bevacizumab + tobemstomig	Resectable disease	Major pathologic response rate	December 2026
NCT04888546	1b/2	Anlotinib + TQB2450	Resectable disease and at high risk of recurrence or metastasis	Pathologic complete response rate, objective response rate	July 2024
NCT05027425	2	Durvalumab + tremelimumab	Listed for liver transplant and within UCSF criteria	Treatment failure	December 2030
NCT04721132	2	Atezolizumab + bevacizumab	Resectable disease	Pathologic complete response rate, treatment-emergent AEs	December 2027
NCT03682276	2	Nivolumab + ipilimumab	Resectable disease	Delay to surgery, treatment-emergent AEs	December 2023
NCT05185739	2	Pembrolizumab + lenvatinib vs pembrolizumab vs lenvatinib	Resectable disease	Major pathologic response rate	July 2026
NCT04615143	2	Tislelizumab ± lenvatinib	Recurrent resectable disease	Disease-free survival	December 2025
NCT03630640	2	Nivolumab, followed by electroporation and adjuvant nivolumab	Eligible for electroporation	1-year local RFS rate	November 2023
NCT04727307	2	Atezolizumab, followed by radiofrequency ablation and adjuvant atezolizumab + bevacizumab vs radiofrequency ablation	Eligible for ablation	RFS	July 2027
NCT05440864	2	Durvalumab + tremelimumab, followed by surgery and adjuvant durvalumab	Resectable disease	Grade ≥ 3 AEs and/or immune-related AEs leading to treatment cessation	November 2026
NCT04930315	2	Camrelizumab + rivoceranib (apatinib), followed by surgery and adjuvant camrelizumab vs adjuvant camrelizumab	Resectable disease	1-year tumor recurrence-free rate	June 2024
NCT04701060	2	Camrelizumab + rivoceranib followed by surgery and adjuvant camrelizumab + rivoceranib	Resectable disease and at high risk of recurrence	Objective response rate	February 2024
NCT05576909	2	Donafenib + TACE followed by surgery and adjuvant donafenib	Listed for liver transplant and outside UCSF criteria	Downstaging success rate	October 2025
NCT03368651	3	Transarterial chemoinfusion with oxaliplatin, calcium folinate, and 5-fluorouracil vs no intervention	Resectable disease with portal vein tumor thrombosis	Overall survival	December 2028
NCT05613478	3	TACE followed by camrelizumab + rivoceranib followed by surgery, adjuvant TACE, and adjuvant camrelizumab + rivoceranib vs adjuvant TACE	Resectable disease	RFS	November 2027
NCT04521153	Unknown	Camrelizumab + rivoceranib followed by surgery, adjuvant TACE, and adjuvant camrelizumab + rivoceranib vs adjuvant TACE	Resectable disease	3-year event-free survival, major pathologic response rate	March 2026
NCT04425226	Unknown	Pembrolizumab + lenvatinib vs no intervention	Listed for liver transplant and exceeding Milan criteria	RFS	December 2024
NCT04443322	Unknown	Durvalumab + lenvatinib	Listed for liver transplant	Progression-free survival, RFS	December 2025

*AE* adverse event, *RFS* recurrence-free survival, *SBRT* stereotactic body radiotherapy, *TACE* transarterial chemoembolization, *UCSF* University of California San Francisco

## Conclusions

The exploration of adjuvant and neoadjuvant treatment for patients with resectable HCC is a rapidly growing field of study. Expansion of liver-directed and systemic treatment options has the potential to provide significant clinical benefit and improve outcomes for this patient population.
